# Aphrodisiac property of aqueous and methanolic extracts of *Raphia vinifera* (Arecaceae) in sexually experienced male rats

**DOI:** 10.18502/ijrm.v17i6.4813

**Published:** 2019-07-29

**Authors:** Pierre Watcho, Fred Lih, Patrick Brice Defo Deeh, Modeste Wankeu-Nya, Esther Ngadjui, Georges Romeo Fozing Bonsou, Albert Kamanyi, Pierre Kamtchouing

**Affiliations:** ^1^Department of Animal Biology, Faculty of Science, Animal Physiology and Phytopharmacology Laboratory, University of Dschang, Dschang, Cameroon.; ^2^Laboratory of Animal Biology and Physiology, Department of Animal Organisms Biology, University of Douala Douala, Cameroon.; ^3^Department of Animal Biology and Physiology, Faculty of Science, University of Yaoundé, Yaoundé, Cameroon.

**Keywords:** Raphia vinifera, Aphrodisiac, Testosterone, Rat.

## Abstract

**Background:**

*Raphia vinifera* (Arecaceae) is a medicinal plant commonly used as a sexual enhancer.

**Objective:**

To investigate the aphrodisiac potential of aqueous extract (AE) and methanolic extract (ME) of *R. vinifera* in sexually experienced male rats.

**Materials and Methods:**

Thirty male Wistar rats were randomly distributed into six groups (5 rats per group) and administered for 14 days with distilled water (10 ml/kg), sildenafil citrate (1.44 mg/kg), and AE or ME of *R. vinifera* (100 or 500 mg/kg). The copulatory activity was tested on days 0, 7, and 14 using receptive females. Further, on day 14, rats were sacrificed and biochemical analyses (testosterone, total protein, and acid phosphatase) were performed.

**Results:**

Sildenafil citrate significantly decreased the intromission latency (day 14, p = 0.04) and frequency (days 7 and 14, p = 0.03) but increased the mount frequency (day 14, p = 0.04), compared with control. Remarkably, *R. vinifera* enhanced the sexual activity by significantly decreasing the intromission latency (AE and ME, 500 mg/kg, day 14, p = 0.04) and increasing the mount frequency (AE and ME, 100 mg/kg, day 7, p = 0.02) compared with control. Moreover, *R. vinifera* improved plasmatic (AE, 100 mg/kg, p = 0.03; AE, 500 mg/kg, p = 0.001; ME, 100 mg/kg, p = 0.01) and testicular (AE, 100 mg/kg, p = 0.001; AE, 500 mg/kg, p = 0.01; ME, 100 mg/kg, p = 0.001; ME, 500 mg/kg, p = 0.01) testosterone levels as well as plasmatic total proteins concentration (ME, 500 mg/kg, p = 0.04).

**Conclusion:**

These findings showed that *R. vinifera* possesses an aphrodisiac property which could further justify its folkloric use in traditional medicine as a sexual enhancer.

## 1. Introduction

An aphrodisiac is defined as any agent capable of arousing the sexual instinct, inducing veneral desire and increasing pleasure and performance (1, 2). Aphrodisiacs are commonly classified into two categories: psycho-physiological stimuli preparations (olfactory, visual, tactile, and aural) and internal agents, mainly obtained from plants (food, alcoholic drinks, and love potion) (1). Male sexual behavior is a complex process coordinated and maintained by internal and external signals (3). It includes precopulatory and copulatory behaviors that permit males to detect and locate females, assess their potential mating appropriateness, thereby stimulating a receptive response. Various synthetic substances such as dopamine, amyl nitrite, and sildenafil citrate, commonly used to improve sexual desire and performance are associated with side effects (4). In developing countries, the search for new compounds from medicinal plants is being intensified, probably due to low side effects, easy availability, low cost, and high efficacy. For instance, *Dracaena arborea* (5), *Guibourtia tessmannii* (6), and *Ficus asperifolia* (7) are reported to have prosexual properties in male rats.

*Raphia vinifera*, commonly known as king bamboo palm is an evergreen, single-stemmed palm tree with an unbranched, rather a stout stem that can be 5-m tall. This plant is found in swamps and other moist locations, especially on the edges of creeks, and distributed from Benin eastward to the Democratic Republic of Congo (8). In West Cameroon, a decoction of the apical bud of *R. vinifera* is used for the treatment of gonorrhea and other genito-urinary infections, while the leaf is used against witchcraft, poison, and various sexually transmitted diseases (9). The oily mesocarp of fruits is eaten and can be fermented into a strong drink for ritual use (9) or to increase sexual ability as per the traditional medicine. The impressive range of medicinal uses of *R. vinifera* fruits may be due to the presence of various phyto-constituents such as alkaloids, flavonoids, saponins, and tannins (10). However, no scientific work has been conducted to confirm its aphrodisiac claim in folkloric medicine.

Therefore, this study was undertaken to evaluate the aphrodisiac potential of *R. vinifera* fruits in sexually experienced male rats. To achieve our goal, mount, intromission, and ejaculatory latencies and frequencies, as well as body and sexual organ weights were evaluated. Testosterone level (plasmatic and testicular), total protein concentration (plasmatic and epidydimal), and prostatic acid phosphatase concentration were also determined. Since the phytochemical compounds revealed in *R. vinifera* extracts act on steroidogenesis (10) by increasing the production of sex hormones, especially testosterone, this plant could be an alternative treatment of erectile dysfunction.

## 2. Materials and Methods

17β-œstradiol benzoate and progesterone (Sigma Chemicals, St Louis, USA), Penicillin G (Clarion Medicals, Nigeria), Diazepam (Renaudin, France), and Ketamine (Rotex Medica, Germany) were used. Estradiol and progesterone were prepared and administered as described previously (7) while other chemicals were directly used. Doses were selected from our previous study (7).

### Plant collection 

Fresh fruits of *R. vinifera* were harvested in March 2014 in Bafou, Menoua Division, west region of Cameroon. It was identified by a botanist (Mr. Victor Nana) at the National Herbarium of Cameroon. After removing the scales of the fruits, they were dried for five days (at room temperature) and reduced to powder. The powder obtained was used for the preparation of the extracts.

### Preparation of the aqueous and methanolic extracts

The powder of *R. vinifera* (100 gr) was macerated in 100 mL of distilled water for 72 hr. The macerate was filtered and the filtrate was oven dried (56°C) to obtain 20.06 g of aqueous extract (AE) of *R. vinifera* (extraction yield: 20.06%). The methanolic extract (ME) was obtained by maceration of 100 gr of powder of *R. vinifera* in 1000 mL of methanol for 72 hr at room temperature. After filtration, the filtrate was evaporated, using a rotary evaporator (75°C) under reduced pressure and 14.50 gr of ME was obtained (extraction yield: 14.5%).

### Animals

Adult male and female Wistar rats (> 90 days, 200–230 gr body weight) were obtained from the animal house of the Department of Animal Biology, Faculty of Science, University of Dschang, Cameroon. Males and females were housed separately and maintained under standard laboratory conditions (12 hr light/dark cycle, 22 ± 1°C), with free access to food and water.

### Experiments

#### Estrus females

The female rats were ovariectomized under anesthesia (diazepam/ketamine). After post-surgical recovery, estrus was induced by the sequential subcutaneous administration of 17β-estradiol benzoate (30 µg) and progesterone (600 µg) after 48 hr and 6 hr, respectively (7). It has been demonstrated that the ovariectomized rat injected with estradiol benzoate has a specific need for copulatory activity with the male rat (11). They were further mated with non-experimental sexually active males and only those exhibiting good sexual receptivity (displayed a high degree of lordosis in response to male's stimulation) and no rejection behavior were selected for the heterosexual copulatory tests.

#### Sexual training and selection of sexually experienced rats

Each male was mated with an estrus female and the sexual behavior-related parameters were recorded as previously described (7). Briefly, single male rat was placed in test cage and an adaptation period (5-10 min) was allowed. An estrus female was introduced in the cage and the copulatory behavior was permitted for 25 min. Male rats exhibiting active sexual behavior (presence of mount and intromission, and ejaculation latencies of less than 15 min) were considered as sexually experienced (7) and used in the present study.

#### Animal repartition and sexual behavior test

Thirty sexually experienced male rats were randomly distributed into six groups (five animals per group) and treated (per os) as follows: group 1, distilled water (10 ml/kg/day); group 2, sildenafil citrate (1.44 mg/kg/day, positive control); groups 3 and 4, AE of *R. vinifera* (100 or 500 mg/kg/day), and groups 5 and 6, ME of *R. vinifera* (100 or 500 mg/kg). All administrations were done orally once a day (between 7:00 am and 8:00 am) for 14 days using a gavage tube. The doses of plant extracts and duration of treatment were chosen from our pilot study (unpublished). On days 0 (before treatment), 7, and 14, the copulatory activity (mount latency, intromission latency, ejaculation latency, mount frequency, intromission frequency, and post-ejaculatory interval) of each male was evaluated in the presence of an estrus female, in a dark and quiet room as described previously (7). Ejaculation was confirmed by the presence of spermatozoa in the vaginal smear of each estrus female at the end of the copulatory test (7). On day 14 after the sexual behavior analysis, plasmatic and testicular testosterone, plasmatic and epidydimal total proteins, as well as prostatic acid phosphatase concentrations were determined.

#### Collection of tissue and organs

Animals were sacrificed by cervical dislocation under anesthesia (diazepam/ketamine). Blood was collected by catheterization of the abdominal artery. Plasma was obtained by centrifugation (3,000× g for 10 min) and stored at –20°C for the biochemical analysis (testosterone, total proteins, and acid phosphatase).

#### Biochemical analysis

Plasmatic testosterone concentration was evaluated using a standard kit (Accubind, Monobind Inc. Lake Forest, USA) according to the manufacturer's instructions. This assay is based on the principle of competitive binding. Competition occurs between unlabeled antigen (present in the samples) and enzyme-labeled antigen (conjugate) for a limited number of antibody binding sites on the microwell plate. After incubation (1 h), the microplate was washed four times and substrate solution was added. (12). Total proteins and acid phosphatase were measured using standard colorimetric kits (CORMAY, Łomianki, Poland) following the procedures outlined in the manufacturer's instructions manual.

### Ethical consideration

The project was presented and validated by the scientific committee of the Department of Animal Biology, University of Dschang, which follows the internationally accepted standard ethical guidelines for laboratory animal use and care as described in the European Economic community guidelines; EEC Directive 86/609/EEC, of the 24th November 1986 (13).

### Statistical analysis 

Data were analyzed by STATISTICA software (version 8.0, StatSoft, Inc., Tulsa, USA). Results were expressed as mean ± SEM (standard error of the mean) and all statistical comparison among groups were done by ANOVA repeated measures followed with post-hoc Tukey HSD test. P-values of less than 0.05 were considered significant.

## 3. Results

### Effects of different treatments on mount, intromission, and ejaculatory latencies 

In all groups, when compared with distilled water or baseline value before treatment (day 0), no statistical change in ML was observed after seven days of treatment. After 14 days, ML was significantly decreased (p = 0.04) in rats given sildenafil citrate compared with distilled water or base line value. In rats treated with AE or ME of *R. vinifera* (500 mg/kg), ML was lowered by –50% and –38%, respectively, compared to the base line value. IL was significantly (p = 0.04) lowered in rats administered with sildenafil citrate (days 7 and 14) and *R. vinifera* (day 14: AE and ME, 500 mg/kg). No significant change in the ejaculatory latency was observed in sildenafil citrate or plant extract-treated rats for 14 days (Table I).

### Effects of different treatments on mount, intromission and ejaculatory frequencies

When compared to the base line value (day 0), sildenafil citrate significantly (p = 0.04) increased the MF after 14 days of treatment. Similarly, the AE (100 mg/kg) and ME (500 mg/kg) of *R. vinifera* improved the sexual performance by significantly increasing (p = 0.02) the MF after seven days of treatment. A trend in increase (77.57 %) of the IF was observed in the rat administered with the AE of *R. vinifera* (100 mg/kg) for seven days. Overall, the efficacy of *R. vinifera* in improving mount and intromission frequencies was more pronounced after 7 days of treatment, while sildenafil citrate exhibited its highest effect after 14 days of treatment (Table II).

### Effects of different treatments on the PEI

The effects of sildenafil citrate and *R. vinifera* extracts on the PEI are presented in Table III. In all animals, no significant change was observed after 7 or 14 days of continuous treatment.

### Effects of different treatments on body and sexual organs weights

**Figure 1:**
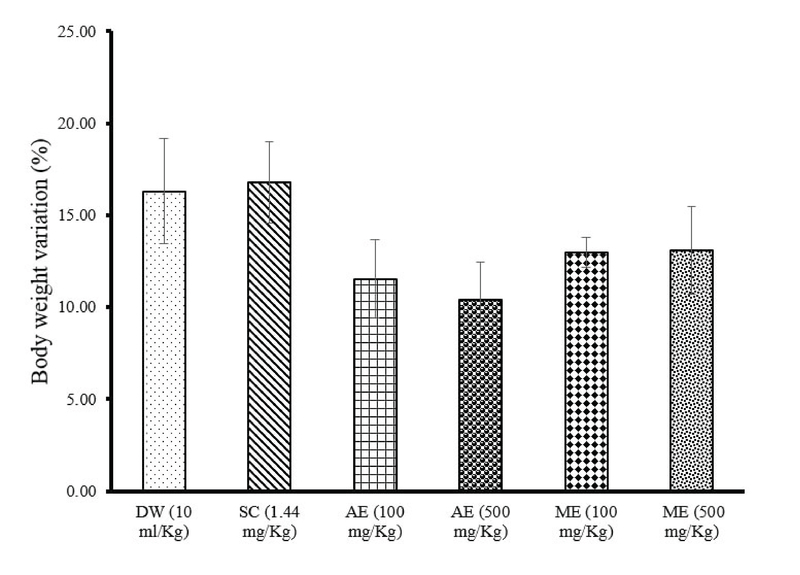
Effects of sildenafil citrate and aqueous extract (AE) or methanolic extract (ME) of *R. vinifera* on body weight after 14 days of treatment. Values were expressed as means ± SEM. Number of rats in each group = 5. DW: Distilled water       SC: Sildenafil citrate       AE: Aqueous extract ME: Methanolic extract

The AE (100 mg/kg and 500 mg/kg) of *R. vinifera* decreased the body weight by -29.11 and -36.32 %, respectively, compared to the control group (Figure 1). In all groups, no statistical difference in testis relative weight was observed, except in rats administered with ME of *R. vinifera* (500 mg/kg, p = 0.04). The relative weight of epididymis showed a significant increase (p = 0.03) in rats administered with the AE of *R. vinifera* (100 mg/kg) compared with control. AE (100 mg/kg) or ME (500 mg/kg) of *R. vinifera* significantly (p = 0.05) increased the vas deferens weight compared with control. In all groups, the relative weight of seminal vesicles and ventral prostate were statistically unchanged (Table IV).

### Effects of different treatments on plasmatic and testicular testosterone levels

**Figure 2:**
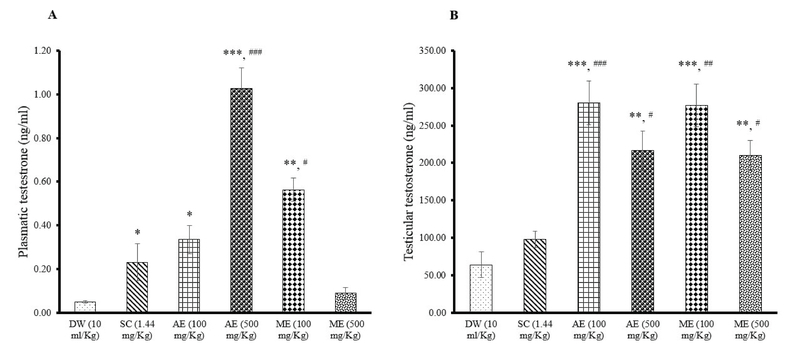
Effects of sildenafil citrate and aqueous extract (AE) or methanolic extract (ME) of *R. vinifera* on plasmatic (A) and testicular (B) testosterone levels after 14 days of treatment. Values were expressed as means ± SEM. Number of rats in each group = 5. *p < 0.05; **p < 0.01; ***p < 0.001: significantly different compared with distilled water. ${}^{\#}$p < 0.05; ${}^{\#\#}$p < 0.01; ${}^{\#\#\#}$p < 0.001: significantly different compared with sildenafil citrate. DW: Distilled water       SC: Sildenafil citrate       AE: Aqueous extract       ME: Methanolic extract

Plasmatic testosterone concentration was significantly elevated in rats administered with sildenafil citrate (p = 0.04; 0.23 ± 0.08 ng/ml) and AE (dose 100 mg/kg; p = 0.03; 0.33 ± 0.06; dose 500 mg/Kg; p = 0.001; 1.03 ± 0.09 ng/ml) or ME (dose 100 mg/kg; p = 0.02; 0.56 ± 0.05 ng/ml) of *R. vinifera*. The highest plasmatic testosterone level was recorded in rats administered with the AE of *R. vinifera* (500 mg/kg) (Figure 2A). When compared to the distilled water (64.09 ± 17.48 ng/ml) or sildenafil citrate (98.15 ± 11.00 ng/ml) groups, testicular testosterone level was found to be significantly increased in rats administered with AE (dose 100 mg/kg; p = 0.001; 280.10 ± 29.07 ng/ml; dose 500 mg/kg; p = 0.01; 216.35 ± 26.17 ng/ml) or ME (dose 100 mg/kg; p = 0.001; 277.14 ± 28.35 ng/ml; dose 500 mg/kg; p = 0.01; 210.10 ± 20.39 ng/ml) of *R. vinifera*. The dose 100 mg/kg of *R. vinifera* exhibited the highest effect on testicular testosterone level (Figure 2B).

### Effects of different treatments on plasmatic and epididymal total protein levels

**Figure 3:**
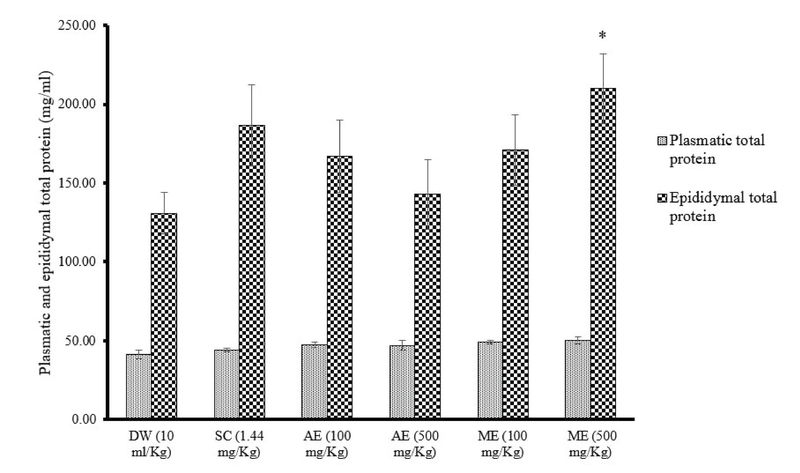
Effects of sildenafil citrate and aqueous extract (AE) or methanolic extract (ME) of *R. vinifera* on plasmatic and epididymal total protein levels after 14 days of treatment. Values were expressed as means ± SEM. Number of rats in each group = 5. *p < 0.05: significantly different compared with distilled water. DW: Distilled water       SC: Sildenafil citrate       AE: Aqueous extract       ME: Methanolic extract

From the results obtained, plasmatic total protein concentration was statistically unchanged in all groups after 14 days of treatment. On the contrary, a significant increase (p = 0.04) in epididymal total protein level was noted in rats administered with ME of *R. vinifera* (500 mg/kg; 209.99 ± 22.09 mg/ml) compared to the control group (130.93 ± 13.31). In the rats treated with sildenafil citrate and AE or ME of *R. vinifera* (100 mg/kg), epididymal total protein level was increased by 42.48, 27.64, and 30.64%, respectively, compared to the distilled water group (Figure 3).

### Effects of different treatments on prostatic acid phosphatase level

**Figure 4:**
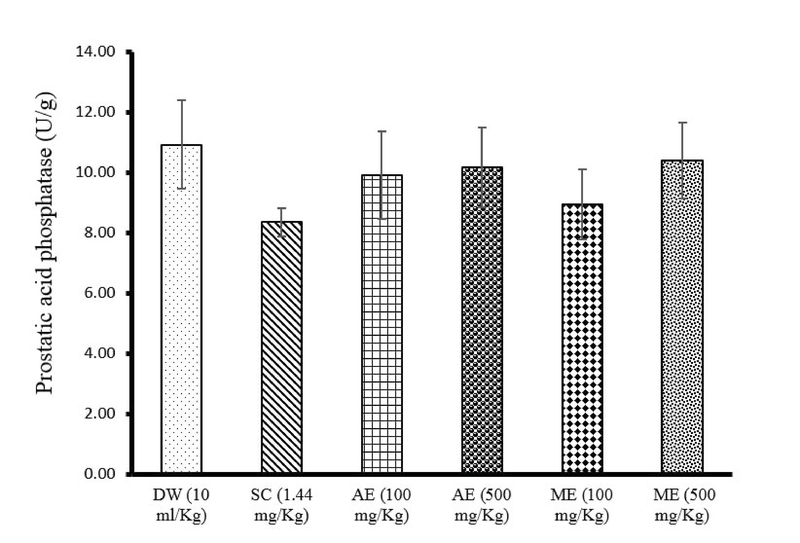
Effects of sildenafil citrate, aqueous extract (AE), or methanolic extract (ME) of *R. vinifera* on prostatic acid phosphatase level after 14 days of treatment. Values were expressed as means ± SEM. Number of rats in each group = 5. DW: Distilled water       SC: Sildenafil citrate       AE: Aqueous extract       ME: Methanolic extract

There was a decrease in prostatic acid phosphatase concentration in rats treated with sildenafil citrate (-23.63%) and AE (100 mg/kg, –9.43%; 500 mg/kg -6.93%) or ME (100 mg/kg, –18.13%; 500 mg/kg -4.85%) of *R. vinifera*. The lowest level of prostatic acid phosphatase concentration was observed in rats treated with sildenafil citrate (8.34 ± 0.46 U/g) (Figure 4).

**Table 1:** Effects of sildenafil citrate and aqueous extract (AE) or methanolic extract (ME) of *R. vinifera* on mount, intromission, and ejaculatory latencies after 7 and 14 days of treatment


**Treatments**	**Mount latency (s)**			**Intromission latency (s)**			**Ejaculatory latency (s)**		
	**Day 0**	**Day 7**	**Day 14**	**Day 0**	**Day 7**	**Day 14**	**Day 0**	**Day 7**	**Day 14**
Distilled water (10 ml/Kg)	21.40 ± 5.57	13.20 ± 2.22	21.20 ± 7.54	26.40 ± 5.57	11.20 ± 2.22	21.40 ± 7.51	406.78 ± 112.92	420.88 ± 125.88	356.10 ± 113.86
Sildenafil citrate (1.44 mg/Kg)	18.33 ± 1.50	7.80 ± 2.27	3.60 ± 1.17*${}^{,\#}$ (p = 0.04)	22.33 ± 1.36	7.80 ± 2.27* (p = 0.04)	3.80 ± 1.11*${}^{,\#}$ (p = 0.04)	487.03 ± 139.53	589.32 ± 125.72	855.52 ± 52.67
AE of *R. vinifera*									
100 mg/kg	20.40 ± 9.73	34.40 ± 11.55	16.60 ± 4.35	29.80 ± 10.22	38.00 ± 12.70	20.40 ± 4.13	398.70 ± 75.08	732.73 ± 118.48	655.07 ± 160.20
500 mg/kg	23.20 ± 5.70	28.80 ± 15.17	11.60 ± 4.70	23.53 ± 5.33	31.40 ± 15.48	11.60 ± 4.70* (p = 0.04)	553.66 ± 80.24	842.03 ± 95.21	537.43 ± 77.48
ME of *R. vinifera*									
100 mg/Kg	23.60 ± 8.49	35.40 ± 16.85	19.67 ± 3.62	32.80 ± 3.09	19.80 ± 7.76	35.27 ± 9.94	571.39 ± 119.15	654.37 ± 153.78	455.44 ± 68.05
500 mg/Kg	20.20 ± 4.72	11.40 ± 4.51	12.60 ± 6.56	20.60 ± 4.62	24.00 ± 10.95	14.00 ± 6.77* (p = 0.04)	502.71 ± 75.65	511.73 ± 142.24	507.03 ± 93.93
white<bcol>10</ecol>Values were expressed as Means ± SEM; number of rats in each group = 5; s: second
white<bcol>10</ecol>*p < 0.05: significantly different compared with distilled water (Day 14); ${}^{\#}$p < 0.05: significantly different compared with sildenafil citrate (Day 0); the numbers in the bracket represent the p-value. ANOVA repeated measure followed with post-hoc Tukey HSD

**Table 2:** Effects of sildenafil citrate and aqueous extract (AE) or methanolic extract (ME) of *R. vinifera* on mount, intromission, and ejaculatory frequencies after 7 or 14 days of treatment


**Treatments**	**Mount frequency**			**Intromission frequency**			**Ejaculatory frequency**		
	**Day 0**	**Day 7**	**Day 14**	**Day 0**	**Day 7**	**Day 14**	**Day 0**	**Day 7**	**Day 14**
Distilled water (10 ml/Kg)	12.23 ± 3.40	12.83 ± 2.12	12.85 ± 2.01	10.83 ± 2.85	11.93 ± 1.81	11.40 ± 1.60	5.00 ± 0.32	3.80 ± 0.49	3.60 ± 0.40
Sildenafil citrate (1.44 mg/Kg)	11.71 ± 2.06	13.04 ± 1.84	17.17 ± 2.75* (p= 0.04)	10.98 ± 1.77	11.64 ± 1.48	15.23 ± 2.46	4.00 ± 0.40	3.40 ± 0.51	3.20 ± 0.58
AE of *R. vinifera*									
100 mg/Kg	11.78 ± 2.13	20.87 ± 1.61* (p = 0.02)	15.52 ± 2.22	10.70 ± 1.53	19.00 ± 2.09	13.88 ± 1.94	4.00 ± 0.63	2.80 ± 0.20	3.40 ± 0.24
500 mg/Kg	17.39 ± 2.74	19.15 ± 3.45	16.33 ± 1.63	13.13 ± 2.60	17.45 ± 2.92	14.03 ± 2.05	3.60 ± 0.40	3.40 ± 0.40	3.00 ± 0.32
ME of *R. vinifera*									
100 mg/Kg	15.17 ± 1.43	16.85 ± 2.15	12.94 ± 1.67	12.99 ± 1.16	15.00 ± 1.80	11.57 ± 1.52	3.60 ± 0.51	3.40 ± 0.40	3.60 ± 0.24
500 mg/Kg	13.53 ± 0.80	21.03 ± 3.62* (p = 0.02)	13.20 ± 1.56	12.00 ± 0.42	17.37 ± 2.64	12.22 ± 1.30	3.80 ± 0.37	3.60 ± 0.24	3.60 ± 0.24
white<bcol>10</ecol>Values were expressed as Means ± SEM. Number of rats in each group = 5; *p < 0.05: significantly different compared with distilled water (Day 0); the numbers in the bracket represent the p-value; ANOVA repeated measure followed with post-hoc Tukey HSD

**Table 3:** Effects of sildenafil citrate and aqueous extract (AE) or methanolic extract (ME) of *R. vinifera* on post-ejaculatory interval after 7 or 14 days of treatment


**Treatments**	**Post-ejaculatory interval(s)**		
	**Day 0**	**Day 7**	**Day 14**
Distilled water (10 ml/Kg)	529.50 ± 90.43	541.75 ± 31.90	527.20 ± 43.08
Sildenafil citrate (1.44 mg/Kg)	570.77 ± 23.60	596.33 ± 100.0	512.87 ± 69.32
AE of *R. vinifera* (100 mg/Kg)	574.28 ± 54.19	490.00 ± 24.94	635.47 ± 17.31
AE of *R. vinifera* (500 mg/Kg)	552.11 ± 27.23	602.73 ± 38.68	537.20 ± 42.93
ME of *R. vinifera* (100 mg/Kg)	612.63 ± 68.29	579.17 ± 38.99	590.40 ± 37.60
ME of *R. vinifera* (500 mg/Kg)	554.17 ± 48.81	577.20 ± 42.68	638.97 ± 19.69
white<bcol>4</ecol>Values were expressed as Means ± SEM; number of rats in each group = 5; s: second

**Table 4:** Effects of sildenafil citrate and aqueous extract (AE) or methanolic extract (ME) of *R. vinifera* on sexual organs weights after 14 days of treatment


**Treatments**	**Relative organs weights (mg/100 g. bw)**				
	**Testis**	**Epididymis**	**Vas deferent**	**Seminal vesicles**	**Ventral prostate**
Distilled water (10 ml/Kg)	1113.40 ± 26.74	356.80 ± 8.13	92.86 ± 4.58	524.70 ± 39.36	145.05 ± 5.37
Sildenafil citrate (1.44 mg/Kg)	1184.87 ± 50.44	384.11 ± 14.59	107.82 ± 4.20	603.84 ± 49.99	188.49 ± 18.95
AE of *R. vinifera* 100 mg/Kg	1082.23 ± 37.54	406.87 ± 14.88* (p = 0.03)	114.66 ± 2.67* (p = 0.05)	586.93 ± 42.40	174.13 ± 6.66
AE of *R. vinifera* 500 mg/Kg	1048.64 ± 41.68	356.47 ± 12.11	102.54 ± 5.53	507.53 ± 36.97	155.49 ± 16.20
ME of *R. vinifera* 100 mg/Kg	1054.75 ± 18.52	370.62 ± 6.54	103.03 ± 5.37	481.06 ± 14.87	174.09 ± 6.05
ME of *R. vinifera* 500 mg/Kg	1156.42 ± 44.73* (p = 0.04)	379.54 ± 15.82	116.94 ± 8.16* (p = 0.05)	521.80 ± 23.96	174.33 ± 13.08
white<bcol>6</ecol>Values were expressed as Means ± SEM; number of rats in each group = 5; *p < 0.05: significantly different compared with distilled water; the numbers in the bracket represent the p-value; ANOVA one-way followed with post-hoc Tukey HSD

## 4. Discussion

The present study conducted in sexually experienced male rats clearly showed that the AE and ME of *R. vinifera* improved the sexual behavior within two weeks. This was achieved by potentiating the sexual drive and performance as evidenced from the reduction in the mount, intromission, and ejaculatory latencies as well as an increase of mount, intromission, and ejaculatory frequencies. Moreover, *R. vinifera* extracts significantly increased plasmatic and testicular testosterone levels as well as epididymal total protein concentration after treatment. These findings indicated that *R. vinifera* is a potential medicinal drug for increasing sexual performances in sexually experienced rats.

Several studies reported that aphrodisiac plants are good alternatives for the improvement of sexual behavior (14, 15), probably due to their efficacy and availability. Aphrodisiacs are agents able to increase libido or sexual response and can be classified (based on their mode of action) into three groups: by amplifying libido, by increasing potency, and by increasing sexual pleasure (16, 17). *R. vinifera* is used in Cameroon traditional medicine to improve sexual performance. In the current study, we investigated the aphrodisiac properties of AE and ME of *R. vinifera* in sexually experienced male rats.

The ML and IL are considered as indicators of sexual motivation (18). The significant reduction in these parameters observed in the rats treated with *R. vinifera* extracts might imply improvement of sexual motivation and sexual appetite which further justifies the folkloric use of this plant as sexual booster. Moreover, the increase of ejaculation latency after treatment with the plant extracts indicates the persistence of sexual drive. On the other hand, MF and IF are the indicators of vigor, libido, and potency. The increase in the MF indicates sexual motivation while elevated IF reflects the efficiency of erection (19). The pro-sexual effect of *R. vinifera* was also established by increasing the MF and IF after treatment. Similar findings have been reported with the AE of *Monsonia angustifolia* (20) and *Acridocarpus smeathmannii* (21). In the current study, the improvement of IL and IF was also observed in rats treated with sildenafil citrate, the most common drug used to treat erectile dysfunction. The beneficial effect of sildenafil citrate on sexual behavior may be due to its steroidogenic potential. Indeed, an inhibition of phosphodiesterase activity during sildenafil therapy significantly increases sex hormone levels including testosterone and potentiates steroidogenesis in Leydig cells (22). The PEI, a good indicator of potency is one of the key parameters evaluated during sexual behavior tests (20). The reduction of PEI reflects an enhancing effect on erectile function and the capacity to have excellent sexual activity (20). In the present study, the decrease of PEI recorded in the rats administered with *R. vinifera* extracts indicates potency and libido improvement.

The AE and ME of *R. vinifera* induced a significant increase in sexual organ weights (testis, epididymis, and vas deferens) after 14 days of treatment. Similar results were found by Besong and colleagues (23) in male rats administered with *Pseudopanax arboreus* extracts. In fact, sexual organ weights are correlated with the production of steroid hormones including testosterone. Since increased sexual organ weights is associated with the genesis of steroid hormones (24), the significant increase in testicular, epididymal, and vas deferens weights observed in rats administered with *R. vinifera* extracts could be due to its androgenic effects. Androgens, especially testosterone, are anabolic compounds capable of improving proteins synthesis and thus muscle weight. Testosterone thereby contributes to the increase of testes, epididymis, and vas deferens weights by stimulating protein synthesis (25). In this study, *R. vinifera* significantly increased the epididymal total protein levels after 14 days of treatment. This increase could be due to the effect of testosterone as reported previously by Gupta and colleagues (26). The increase in testosterone in animals treated with *R. vinifera* could be due to a direct action of its active components on the gonadal tissues or on the hypothalamic-pituitary-testis axis. Similar results were reported by Chung and colleagues (27). Prostatic acid phosphatase, a glycoprotein which indicates prostate activity (28), was lowered in the rats treated with *R. vinifera* extracts. This decrease in prostatic acid phosphatase concentration after *R. vinifera* application could probably be attributed to the improvement in testosterone level. These findings corroborated with those reported by Watcho and colleagues (6) after *Ficus asperifolia* treatment.

The aphrodisiac property of *R. vinifera* could be due to the various active components present in this plant. Previous phytochemical analysis of *R. vinifera* revealed the presence of saponins, terpenoids, and flavonoids (10). It has been reported that flavonoids facilitate male sexual behavior by boosting testosterone production and/or preventing its metabolic degradation (29). Terpenoids have an implication in the triggering of penile erection as well as in the improvement of the sexual performances (30). Kim and colleagues (31) demonstrated that saponin facilitated relaxation of the corpus cavernosum muscles by stimulating the L-arginine/nitric oxide pathway. These bioactive components could have an effect on the central nervous system by activating neurotransmitters or the periphery by stimulating the release of nitric oxide (14).

## 5. Conclusion

In conclusion, *R. vinifera* boosted testosterone production, improved protein synthesis, and enhanced the copulatory activity in sexually experienced male rats. These findings further justify the folkloric use of *R. vinifera* as a sexual enhancer.

##  Conflict of Interest 

The authors declare that they have no conflict of interest.
